# Increased incidence of bladder cancer with metabolically unhealthy status: analysis from the National Health Checkup database in Korea

**DOI:** 10.1038/s41598-020-63595-3

**Published:** 2020-04-15

**Authors:** Jong Wook Kim, Sun Tae Ahn, Mi Mi Oh, Du Geon Moon, Jun Cheon, Kyungdo Han, Seon Mee Kim, Hong Seok Park

**Affiliations:** 1 Department of Urology, Korea University College of Medicine, Seoul, Republic of Korea; 2 Department of Biostatistics, College of Medicine, The Catholic University of Korea, Seoul, Republic of Korea; 3 Department of Family Medicine, Korea University College of Medicine, Seoul, Republic of Korea

**Keywords:** Cancer epidemiology, Bladder cancer, Bladder

## Abstract

We assessed the association between metabolic health status and the incidence of bladder cancer using nationally representative data from the National Health Insurance System and National Health Checkups (NHC) databases in South Korea. Data for 11,781,768 men who participated in the NHC between 2009 and 2012 were analysed. The normal-weight and physically obese categories were defined as body mass indexes (BMI) < 25 and ≥25 kg/m^2^, respectively. Metabolically obese was defined as the presence of ≥3 components of metabolic syndrome. The participants were stratified into metabolically healthy, normal-weight (MHNW); metabolically obese, normal-weight (MONW); metabolically healthy, obese (MHO); metabolically obese, obese (MOO). Multivariate-adjusted Cox regression analysis was conducted to examine the association between metabolic health status and the incidence of bladder cancer. The study participants included 17,777 men newly registered with bladder cancer. Analysis according to metabolic health status classification revealed a higher multivariable-adjusted hazard ratio in the MOO, MONW group than in the MHO group (1.307 [95% CI: 1.258–1.358], 1.183 [95% CI: 1.137–1.231] and 1.066 [95% CI: 1.017–1.119], respectively; hazard ratios given relative to MHNW group) We found an association between metabolic health status and the incidence of bladder cancer, with an increasing risk according to the number of metabolic health status components.

## Introduction

Obesity is associated with diabetes, cardiovascular disease, several types of cancers, and a decreased life expectancy^1^. However, not all obese patients are at high risk for these diseases, indicating that there are ‘healthy obese’ individuals among obese patients. Recently, metabolically healthy obese (MHO) patients have been defined as those with increased body weight and body mass index (BMI) but no or few metabolic conditions such as diabetes, hypertension, and dyslipidaemia^2,3^. Conversely, the metabolically obese, normal-weight (MONW) patients, defined as those with a normal body weight but various metabolic diseases, have a high risk for various diseases and a high mortality rate^4–8^.

The existing studies on cancer epidemiology are mostly on the effects of obesity or metabolic syndrome on cancer incidence; however, this subject has not been sufficiently explored with regard to the metabolic health status.

Bladder cancer is one of the ten most common cancers. Each year, there are 380,000 new patients, among whom 150,000 die^9,10^.

There have been studies on the relationship between obesity and bladder cancer^11,12^ and between metabolic syndrome and bladder cancer^13,14^, but no study has been conducted on the relationship between bladder cancer and metabolic health. It is unknown whether bladder cancer risk is different for metabolically healthy obese and metabolically obese normal weight men.

In this study, we aimed to assess the association between metabolic health status and the incidence of bladder cancer using nationally representative data of the Korean population.

## Materials and Methods

### Data source and study population

In South Korea, the National Health Insurance System (NHIS) established in 2000 covers about 98% of the citizens (about 50 million in 2014)^15^. All insurance members and dependents are asked to undergo a free biannual health check-up as part of the NHIS. In 2013, about 68% of members received a full health check-up^16^, making it possible to obtain information on population-wide disease status^17^. In the present study, sex, birth date, past history, medication history, socioeconomic data, body size phenotypes, and diagnostic codes based on the International Statistical Classification of Diseases and related health problems 10th revision, Clinical Modification (ICD-10-CM) were retrieved.

Data from a total of 11,781,768 men who participated in the National Health Checkups (NHC) program between January 2009 and December 2012 were analysed. Body size phenotypes were classified according to BMI, which was calculated from measured height and weight, and the presence or absence of metabolic syndrome according to the results of the NHC^17^.

**Figure 1:**
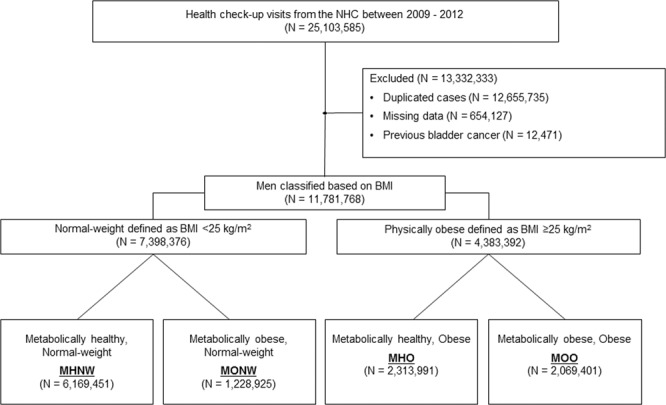
Flow chart of the study population.

Since patients that are diagnosed with cancer, cardiovascular disease, cerebrovascular disease, and rare disease are entitled to economic benefits from the Korean government^18^, almost all physician-diagnosed cancer patients are registered in the central database^19^. Bladder cancer was coded as C67 in the ICD-10-CM. A flowchart for the selection of the study participants is shown in Fig. 1.

### Definition of metabolic health status

‘Physically obese’ was categorized according to the World Health Organization’s (WHO) definition of obesity for Asians. The study population was divided into the (physically) obese (BMI ≥ 25 kg/m^2^) and normal weight (BMI < 25 kg/m^2^) groups^20,21^.

‘Metabolically obese’ was defined according to the modified criteria by the National Cholesterol Education Program-Adult Treatment Panel III. This criteria requires the presence of three or more of the following components: triglyceride level ≥150 mg⁄dL, high-density lipoprotein (HDL) cholesterol level <40 mg ⁄ dL, fasting glucose level ≥100 mg⁄ dL (or taking anti-diabetic medications), blood pressure (BP) ≥ 130⁄85 mmHg (or taking antihypertensive drugs), or waist circumference (WC) ≥ 90 cm, according to the Asian-specific WC cut-off^22,23^.

Each metabolic disease was defined based on the health check-up data. Systolic and diastolic BP measurements were taken in a seated position after 5 minutes of rest. Blood sampling for glucose and lipid profile was performed after overnight fasting^19^. Smoking, drinking, exercise, and socio-economic status data were self-reported.

Subjects were stratified into four groups based on their metabolic health status: metabolically healthy, normal-weight (MHNW); MONW; MHO; and metabolically obese, obese (MOO).

### Statistical analyses

Data were analysed using SAS version 9.3 (SAS Institute, Cary, NC, USA). All values in this study were expressed as the mean ± standard deviations (SD) and proportions for continuous and categorical variables. A multivariate-adjusted Cox regression analysis was performed to assess the hazard ratios (HRs) and 95% confidence intervals (CIs) for the association between bladder cancer and metabolic health status. Age, smoking status, alcohol consumption, exercise, and economic status were adjusted. A P-values below 0.001 was considered significant.

### Ethical statement

The present study protocol was reviewed and approved by the Institutional Review Board (IRB) of the Korea University Guro Hospital. The requirement for informed consent from the participants was waived by the IRB (No. 2017GR0219). The present study was performed in accordance with relevant guidelines and regulations. To protect the individuals’ privacy, each patient’s identification number was anonymized.

## Results

### Baseline characteristics of the study population

This study included men who received an NHC between 2009 and 2012. For those who received an NHC two or more times in the above period, only the results from the first check-up were included. In addition, 12,471 subjects who were previously diagnosed with bladder cancer were excluded. In the end, of the 25,103,585 possible check-up visits, 11,781,768 were enrolled in this study (Fig. 1). Mean age was 46.5 ± 13.6 years, mean BMI was 24.2 ± 1.9 kg/m^2^ and mean WC was 83.7 ± 5.8 cm. The median follow-up duration was 5.4 ± 1.1 years. Among them, 10.7% had incident diabetes, 28.0% had incident hypertension and 17.8% had incident dyslipidaemia.

The participants were classified according to BMI, with 7,398,376 (62.7%) in the normal-weight group and 4,383,392 (37.3%) in the obese group.

Comparison between these two groups revealed significant differences in age distribution, smoking, drinking, exercise, diabetes, hypertension, BMI, WC, BP, glucose level, and lipid profile (P < 0.001).

The two groups were then divided into four groups according to metabolic health status.

The MHNW group included 6,169,451 men (52.4%), the MONW group included 1,228,925 (10.4%), the MHO group included 2,313,991 (19.6%), and the MOO group included 2,069,401 (17.6%).

Between these groups, there were significant differences in age, WC, BP, fasting glucose level, and lipid profile. In the MONW group, 30.7% had incident diabetes, 58.2% had incident hypertension, and 46.3% had incident dyslipidaemia. In the MHO group, 3.9% had incident diabetes, 19.7% had incident hypertension, and 11.8% had incident dyslipidaemia.

The mean age was highest in the MONW group, followed by the MOO, MHNW, and MHO groups. In terms of lifestyle, the MHNW group had the highest smoking rate, while the MOO group showed the highest rate of frequent drinking (more than once per week). The rate of regular exercise (once per week) was lowest in the MONW group.

Table 1 summarizes the general characteristics of the study population and subgroups.

**Table 1:** Baseline Characteristics of Subjects According to BMI and Metabolic Health Status.

Group	Normal-weight		Obese	
	MH	MO	MH	MO
n	6,169,451	1,228,925	2,313,991	2,069,401
Age, years	44.9 ± 14.4	55.6 ± 12.7	43.2 ± 12.5	49.6 ± 12.7
<40	2,453,187 (39.76)	133,158 (10.84)	981,738 (42.43)	472,895 (22.85)
40–65	3,029,928 (49.11)	775,960 (63.14)	1,188,952 (51.38)	1,315,890 (63.59)
>65	686,336 (11.12)	319,807 (26.02)	143,301 (6.19)	280,616 (13.56)
Smoking (current)	2,863,350 (46.41)	514,112 (41.83)	995,566 (43.02)	867,794 (41.93)
Drinking (>30 g/day)	649,333 (10.52)	166,209 (13.52)	284,874 (12.31)	321,987 (15.56)
Exercise (regularly)	3,379,980 (54.79)	636,449 (51.79)	1,397,338 (60.39)	1,155,847 (55.85)
Diabetes	292,676 (4.74)	376,774 (30.66)	90,787 (3.92)	504,086 (24.36)
Hypertension	960,563 (15.57)	715,402 (58.21)	455,551 (19.69)	1,169,163 (56.5)
Dyslipidaemia	476,703 (7.73)	569,122 (46.31)	273,157 (11.8)	788,176 (38.09)
BMI	22.17 ± 1.89	23.05 ± 1.57	26.82 ± 1.7	27.8 ± 2.24
WC, cm	79.07 ± 5.82	83.76 ± 5.92	87.83 ± 5.62	92.78 ± 6.12
Systolic BP, mmHg	121.15 ± 13.23	131.22 ± 14.52	123.96 ± 12.77	132.34 ± 13.95
Diastolic BP, mmHg	75.72 ± 9.13	81.03 ± 9.94	77.88 ± 9.08	82.75 ± 9.92
Fasting glucose, mg/dL	94.28 ± 19.4	116.24 ± 36.66	94.2 ± 16.78	111.84 ± 31.77
Total cholesterol, mg/dL	188.53 ± 33.63	196.23 ± 42.35	198.41 ± 34.06	202.5 ± 39.96
HDL, mg/dL	55.3 ± 15.8	48.21 ± 16.76	51.84 ± 14.76	46.7 ± 15.34
LDL, mg/dL	109.82 ± 30.99	108.29 ± 38.81	118.46 ± 31.61	112.77 ± 37.08
GFR, mL/min/m^2^	91.77 ± 46.24	86.25 ± 39.42	90.29 ± 48.44	87.02 ± 41.98

Abbreviations: MH, metabolically healthy; MO, metabolically obese; BMI, body mass index; WC, waist circumference; BP, blood pressure; HDL, high-density lipoprotein; LDL, low-density lipoprotein GFR, glomerular filtration rate.

Data were expressed as the mean ± standard deviations (SD).

Current smoking: continuing smoking and smoked ≥100 cigarettes in lifetime.

Drinking: drinking ≥30 g of alcohol per day.

Exercise: doing exercise more than 20 minutes ≥1 time per week.

Dyslipidaemia: triglyceride level ≥150 mg⁄dL, high-density lipoprotein cholesterol level <40 mg ⁄ dL.

Diabetes: fasting glucose level ≥100 mg⁄ dL or taking anti-diabetic medications.

Hypertension: blood pressure ≥130⁄85 mmHg or taking antihypertensive medications.

### Incidence and risk of bladder cancer according to obesity and metabolic health status

Within the 5.4 ± 1.1 years of follow-up, 17,777 of the patients were newly-diagnosed and registered with bladder cancer.

After dividing the groups according to BMI levels, multivariable (age, smoke, drinking, exercise)- adjusted HR for the incidence of bladder cancer was the lowest in the group with a BMI < 18.5 kg/m^2^ and highest for those with a BMI between 25–30 kg/m^2^. When the subjects were divided according to WC, hypertension, diabetes, high triglyceride, and low high-density lipoprotein, the HRs were 1.217 (95% CI: 1.178–1.256), 1.106 (95% CI: 1.071–1.142), 1.12 (95% CI: 1.087–1.154), 1.178 (95% CI: 1.144–1.213), and 1.199 (95% CI: 1.162–1.237), respectively (Table 2).

**Table 2:** Association between Metabolic Parameters and Incidence Rate of Bladder Cancer.

	n	Event	Duration (person-years)	IR (per 1000 person-years)	Multivariable-adjusted HR * (95% CI)
BMI, kg/m^2^					
<18.5	280,457	573	1,451,988.41	0.39463	0.968 (0.889–1.055)
18.5–23	3,970,603	6,052	21,405,151.08	0.28274	1 (ref.)
23–25	3,147,316	4,914	17,099,066.95	0.28738	1.085 (1.045–1.127)
25–30	3,934,367	5,825	21,279,792.75	0.27373	1.156 (1.115–1.199)
>30	449,025	413	2,374,578.85	0.17393	1.146 (1.037–1.267)
WC, cm					
<90	9,155,550	12,411	49,532,933.98	0.25056	1 (ref.)
≥90	2,626,218	5,366	14,077,644.07	0.38117	1.217 (1.178–1.256)
HTN					
No	5,942,325	5,647	32,204,726.79	0.17535	1 (ref.)
Yes	5,839,443	12,130	31,405,851.26	0.38623	1.106 (1.071–1.142)
DM					
No	7,487,275	8,976	40,615,361.53	0.221	1 (ref.)
Yes	4,294,493	8,801	22,995,216.52	0.38273	1.12 (1.087–1.154)
High TG					
No	6,848,357	9,372	36,955,739	0.2536	1 (ref.)
Yes	4,933,411	8,405	26,654,839.05	0.31533	1.178 (1.144–1.213)
Low HDL					
No	9,180,364	11,784	49,671,734.08	0.23724	1 (ref.)
Yes	2,601,404	5,993	13,938,843.97	0.42995	1.199 (1.162–1.237)
MO					
No	8,483,442	10,151	45,964,233.99	0.22085	1 (ref.)
Yes	3,298,326	7,626	17,646,344.06	0.43216	1.224 (1.188–1.261)

Abbreviations: IR, incidence rate; HR, hazard ratio; CI, confidence interval; BMI, body mass index; WC, waist circumference; HTN, hypertension; DM, diabetes mellitus; TG, triglyceride; LDL, low-density lipoprotein; MO, metabolically obese.

*Adjusted for age, smoking, alcohol drinking, exercise, and income.

**Figure 2:**
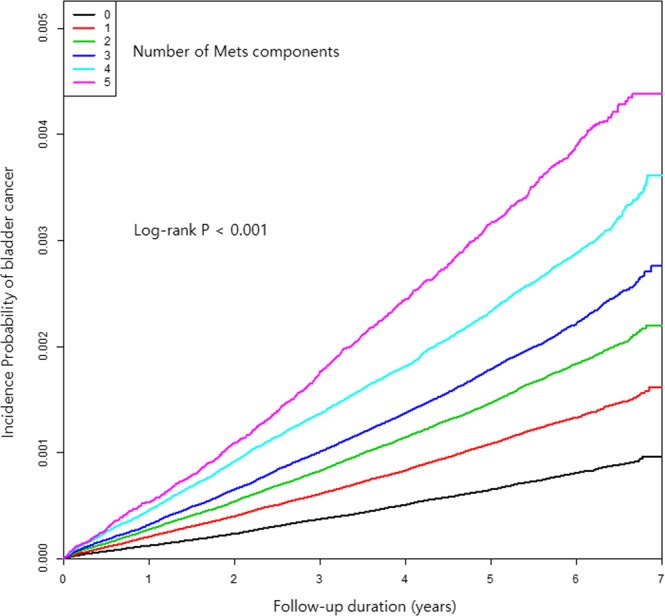
Kaplan-Meier estimates of survival curves for the time to incident bladder cancer, stratified by metabolic health status. The median follow-up duration was 5.4 years. The subjects were divided into six groups according to the number of components of metabolic health status (log-rank test, P < 0.001).

Of the 3,298,326 metabolically obese patients, 7,626 were newly-diagnosed with bladder cancer, resulting in an HR of 1.224 (95% CI: 1.188–1.261). As the number of metabolic factors increased, the HR also increased significantly (Fig. 2).

After the subjects were stratified into the MHNW, MONW, MHO, and MOO groups, multivariable-adjusted HRs were 1.183 (95% CI: 1.137–1.231) in the MONW group, 1.066 (95% CI: 1.017–1.119) in the MHO group, and 1.307 (95% CI: 1.258–1.358) in the MOO group, showing a higher HR in the MONW group than in the MHO group. In additional secondary analysis excluding the first 12 months, the results remain unchanged. (Table 3)

**Table 3:** Association between Metabolic Status and Incidence of Bladder Cancer.

	group	n	Events	Duration	IR (per 1000 person-years)	HR (95% C.I.)	
						Age-adjusted	Multivariable-adjusted *
Total	MHNW	6,169,451	7,958	33,409,087.62	0.2382	1 (ref.)	1 (ref.)
	MONW	1,228,925	3,581	6,547,118.82	0.54696	1.187 (1.14–1.235)	1.183 (1.137–1.231)
	MHO	2,313,991	2,193	12,555,146.37	0.17467	1.024 (0.976–1.073)	1.066 (1.017–1.119)
	MOO	2,069,401	4,045	11,099,225.24	0.36444	1.271 (1.224–1.32)	1.307 (1.258–1.358)
Excluding first 12 mo	MHNW	6,152,554	6,583	27,245,622.8	0.24162	1 (ref.)	1 (ref.)
	MONW	1,221,859	2,950	5,320,706.37	0.55444	1.189 (1.138,1.242)	1.185 (1.135,1.238)
	MHO	2,311,069	1,851	10,242,217.94	0.18072	1.036 (0.983,1.091)	1.078 (1.023,1.135)
	MOO	2,064,694	3,403	9,031,526.33	0.37679	1.289 (1.237,1.344)	1.324 (1.27,1.38)

Abbreviations: IR, incidence rate; HR, hazard ratio; CI, confidence interval; MHNW, metabolically healthy, normal-weight, MONW; metabolically obese, normal-weight; MHO, metabolically healthy, obese; MOO, metabolically obese, obese.

*Adjusted for age, smoking, alcohol drinking, exercise, and income.

Among patients 65 years of age and older, multivariable HR of the MHO group was 0.923 (95% CI: 0.789–1.079), lower than that of the MHNW group. Among nonsmokers, multivariable-adjusted HRs were 1.186 (95% CI: 1.127–1.248) in the MONW group, 1.081 (95% CI: 1.019–1.148) in the MHO group, and 1.33 (95% CI: 1.269–1.395) in the MOO group, showing a higher HR in the MONW group than in the MHO group (Table 4).

**Table 4:** Subgroup analysis according to age and smoking.

	group	n	Events	Duration	IR (per 1000 person-years)	HR (95% C.I.)	
						Age-adjusted	Multivariable-adjusted *
Age <65 y	MHNW	5,483,115	3,477	29,793,347.87	0.1167	1 (ref.)	1 (ref.)
	MONW	909,118	1,358	4,890,175.63	0.2777	1.24 (1.173,1.31)	1.271 (1.203,1.343)
	MHO	2,170,690	1,287	11,775,367.33	0.1093	1.023 (0.959,1.09)	1.067 (1.001,1.138)
	MOO	1,788,785	1,984	9,605,029.14	0.20656	1.237 (1.161,1.318)	1.222 (1.147,1.302)
Age ≥65 y	MHNW	686,336	4,481	3,615,739.75	1.2393	1 (ref.)	1 (ref.)
	MONW	319,807	2,223	1,656,943.19	1.34163	1.233 (1.088,1.398)	1.23 (1.085,1.394)
	MHO	143,301	906	779,779.04	1.16187	0.917 (0.785,1.072)	0.923 (0.789,1.079)
	MOO	280,616	2,061	1,494,196.1	1.37934	1.245 (1.089,1.422)	1.238 (1.083,1.414)
Nonsmokers	MHNW	3,306,101	4,615	17984042.56	0.25662	1 (ref.)	1 (ref.)
	MONW	714,813	2,233	3827455.02	0.58342	1.202 (1.143,1.265)	1.186(1.127,1.248)
	MHO	1,318,425	1,453	7184763.65	0.20223	1.084(1.021,1.15)	1.081(1.019,1.148)
	MOO	1,201,607	2,744	6475371.99	0.42376	1.345(1.283,1.41)	1.33(1.269,1.395)

Abbreviations: IR, incidence rate; HR, hazard ratio; CI, confidence interval; MHNW, metabolically healthy, normal-weight, MONW; metabolically obese, normal-weight; MHO, metabolically healthy, obese; MOO, metabolically obese, obese.

*Adjusted for age, smoking, alcohol drinking, exercise, and income.

## Discussion

In this study, we confirmed a significant correlation between metabolic health status and the incidence of bladder cancer.

First, after we corrected for age and various other factors, we found the HR for the incidence of bladder cancer was highest in the MOO group, followed by the MONW, MHO, and MHNW groups. Second, the HR increased with an increasing number of factors related to metabolic health status.

Although BMI is generally used to define obesity, this measure cannot assess the distribution of fat and muscle. Therefore, obesity determined on BMI is not enough to judge its effect on cancer. There are many studies on obesity and cancer, but the results of these studies need to be interpreted carefully.

More important than BMI is the presence of metabolic disease. In this study, the HR of the MONW patients was higher than that of the MHO patients, suggesting that metabolic health status is a more important factor than BMI-based obesity in terms of bladder cancer.

The definition of metabolic health has not yet been established. In general, good metabolic health is defined as the absence of metabolic disorders such as type 2 diabetes, dyslipidaemia, and hypertension^24^.

We defined the metabolically obese as having ≥3 components of metabolic syndrome according to the definition proposed by Meigs *et al*.^7^, which is the same as the definition of metabolic syndrome in the NCEP-ATP III.

Metabolic obesity is regarded as a combination of central obesity, diabetes, hypertension, and dyslipidaemia, and each of these components can affect the development of bladder cancer.

According to the prospective cohort study by Haggstrom *et al*. involving 580,000 subjects^14^, metabolic syndrome significantly increased the risk of bladder cancer in men (relative risk [RR] = 1.10; 95% CI: 1.01–1.18).

In a meta-analysis, Esposito *et al*.^13^ also reported that metabolic syndrome was associated with an increased risk of bladder cancer in men. However, since metabolic syndrome is a combination of various diseases, some people argue that analysing it as a sole condition is not appropriate^25^.

To date, there is no definite conclusion regarding the relationship between obesity and bladder cancer.

Holick *et al*.^11^ and Haggstrom *et al*.^14^ reported no correlation between obesity and bladder cancer. Additionally, the International Agency for Research on Cancer reported that although colon, stomach, liver, gallbladder, pancreas, kidney cancer had a relationship with obesity, there was no evidence that bladder cancer was associated with obesity^26^.

However, Koebnick *et al*.^12^ claimed that obesity increased the risk of bladder cancer and Ferro *et al*.^27^ revealed that BMI plays a role in the recurrence and progression of non-muscle-invasive bladder cancer. In the Asian population, a recent study^28^ showed that the incidence of bladder cancer was higher in the overweight and obese population.

In one study conducted in Asia, Xu *et al*.^29^ reported that diabetes and hypertriglyceridemia but not obesity and hypertension increased the risk of bladder cancer; as the number of metabolic syndrome components increased, so did the risk of bladder cancer.

Abdominal obesity has been proposed to better reflect obesity-related health risk than obesity based on BMI^30^. Montella *et al*.^31^ reported that abdominal obesity increased the risk of bladder cancer by 63% (95% CI: 1.22–2.19). Cantiello *et al*.^32^ revealed that visceral obesity was a predictor and adverse pathological feature in bladder cancer. In this study, a metabolically obese status had a more significant influence on bladder cancer incidence than physical obesity.

There are recent studies on the relationship between systemic inflammation markers and cancer outcomes^33^. Systemic inflammatory status is also suggested as a possible predictor of diabetes, obesity, and cancer. Unfortunately, we could not check the inflammatory status in this study.

One strength of this study was the nationwide large-scale sample size, which allows the results to be generally applied to Korean or East Asian populations. A considerable amount of the Korean population receives NHCs. As most cancer patients receive C codes through national registration, this study has high reliability compared to that of other cohort studies. Even though the differences in HR were not large, all of them were statistically significant due to the large number of subjects.

This study has several limitations. First, the data lack detailed information on bladder cancer; therefore, research on the cancer stage and histological differences was not possible. Only the incidence could be analysed and relapse or mortality rates could not be assessed. Second, for the same reason, we could not perform subgroup analysis for smoking, exercise, and alcohol intake. As smoking is one of the strongest known risk factors for bladder cancer, variable smoking status or pack-years data is important for studying bladder cancer. Additionally, since this was a huge dataset, we could not perform a quantitative analysis. Third, there was a risk of selection bias because health check-ups are attended mostly by healthy people. Fourth, the groups could change with time, however, we could not perform a time-dependent analysis. We checked and stratified the groups only at the time of enrollment.

Despite these limitations, to our knowledge, our study is the first large-scale cohort study to show the relationship between metabolic health status and bladder cancer. In the future, extensive research will be necessary along with more detailed clinical patient data.

## Conclusion

This large-scale cohort study showed a significant relationship between metabolic health status and the incidence of bladder cancer. The risk increased with the number of metabolic health components. The hazard ratio for the incidence of bladder cancer was higher in MONW patients than in MHO patients.
